# Efficacy of diet on fatigue, quality of life and disability status in multiple sclerosis patients: rapid review and meta-analysis of randomized controlled trials

**DOI:** 10.1186/s12883-022-02913-w

**Published:** 2022-10-20

**Authors:** María Dolores Guerrero Aznar, María Dolores Villanueva Guerrero, Jaime Cordero Ramos, Sara Eichau Madueño, María Morales Bravo, Rocío López Ruiz, Margarita Beltrán García

**Affiliations:** 1 Pharmacy Clinical Management Unit, Virgen Macarena University Hospital, Seville, Spain; 2 Neurology Clinical Management Unit, Virgen Macarena University Hospital, Seville, Spain

**Keywords:** Meta-analysis, Diet, Multiple sclerosis, EDSS, MFIS, Quality of life

## Abstract

**Background:**

Multiple sclerosis is an inflammatory and neurodegenerative disease. People with multiple sclerosis (pwMS) experience chronic fatigue which is difficult to deal with therapeutically and greatly affects health-related quality of life (QOL).

PwMS are aware of the lack of generalized dietary advice related to their disease, leading to self-experimentation with diet.

It is necessary to provide objective information about dietary interventions for pwMS. We aim to provide an objective synthesis of the evidence for efficacy and safety of specific diets in pwMS through a rapid review and meta-analyses of randomized controlled trials (RCTs), examining symptomatic fatigue (MFIS), QOL, Expanded-Disability-Status-Scale (EDSS), and severe adverse events.

**Methods:**

We have carried out a rapid review (MEDLINE and EMBASE) up to December 2021, with PRISMA methodology, and meta-analyses, of (RCTs). All statistical analyses were performed using the comprehensive meta-analysis (CMA) -RStudio 4.1.3. The analysis used weighted mean differences (WMD) and a 95% confidence interval (CI) using a random-effects model to compare the effects of the dietary intervention with the control.

**Results:**

Eight studies met the inclusion criteria. Of these eight studies, five analyzed EDSS, three MFIS, and three QOL. A total of 515 patients were analyzed. These meta-analyses cumulative evidence support that dietary intervention is associated with a trend of reduction in fatigue (308 patients studied) -the difference between means (SMD) of the control group and intervention group was -2,033, 95%-IC (-3,195, -0,152), a *p*-value of 0.0341)-, an increase in QOL (77 patients studied), no significant effect on EDSS (337 patients studied), and no severe adverse events.

**Conclusions:**

It is difficult to reach a high level of evidence in dietary studies. Our findings show that dietary intervention is associated with a trend of reduction in fatigue in MS. Taking into account the potential of dietary interventions and the benefit/risk ratio in their favor, neurologists must be aware of the great importance of making interventions on diet in MS if necessary. There are dietary interventions with some evidence of benefit for patients with MS, which could be chosen based on adherence, patient preferences, and individual outcomes. Large prospective clinical trials are needed to shed further light on this topic.

**Supplementary Information:**

The online version contains supplementary material available at 10.1186/s12883-022-02913-w.

## Background

Multiple sclerosis (MS) is an inflammatory, demyelinating and neurodegenerative disease of the central nervous system. There are several hypotheses correlating inflammation and neurodegeneration in MS. Differences in the onset of lesions, either by axonal loss or demyelination together with the proportions of inflammation, demyelination/remyelination, and neurodegeneration in each patient could explain the different disease courses described [1]. Multiple sclerosis can be classified according to three phenotypes: relapsing–remitting disease (RRMS), primary progressive disease (PPD), and secondary progressive disease (SPD). Descriptions of the clinical course of multiple sclerosis include disease activity and progression [2]. Depression and fatigue are often considered the predictor variables with the greatest weight in changes in quality of life (QOL) in MS patients [3].

### 1. Why it is important to do a rapid review on diet-related evidence outcomes MS patient-focused?

First, there is a lack of scientific consensus on dietary and nutritional recommendations for MS. A review published by Cochrane in 2020 concludes that there is insufficient evidence to support specific dietary and nutritional interventions in multiple sclerosis [4].

Nutrition is considered a possible cofactor influencing the inflammatory cascade. It influences both the molecular level and the gut microbiota. The nutritional status and dietary habits of patients with MS are not often studied and the lack of a consensus on dietary recommendations may lead many patients to try alternative dietary regimens without any scientific basis, increasing the risk of malnutrition [5].

Although people with MS often use the Internet to obtain information about the pathology and possible therapeutic alternatives such as diet, neurologists remain their preferred source of consultation [6]. There are many websites that offer advice, suggestions, and dietary regimens as a basis for managing symptomatology and even disease progression. In contrast, very little of this information freely published on the Web is supported by scientific evidence [7].

Surveys reported by Yadav et al. (2010) show that between 30 and 60% of MS patients use or have used complementary medicine therapies and supplements. This study reflects that the most commonly used interventions with the greatest positive impact are dietary modifications, omega-3 fatty acid supplementation, and antioxidants [8].

#### Persistent gastrointestinal symptoms in many MS patients

Patients with MS often develop gastrointestinal (GI) symptoms on a regular basis. Levinthal et al. show in their study that almost 65.6% of MS patients have at least one persistent GI symptom (dysphagia, dyspepsia, constipation, and/or inflammatory bowel disease (IBD) [9].

One systematic review based on case–control studies appears to find an association of a 50% higher relative risk of developing Inflammatory bowel disease (IBD) or MS if you already have MS or IBD respectively [10]. In a retrospective study of medical records, the observed prevalence of MS at the onset of IBD was 3.7 times higher than expected [11].

#### Role of the intestinal microbiota in MS

The gut microbiota is defined as the sum of all microorganisms housed in the gut, including their genes, proteins, and metabolic products. The gut microbiota has recently emerged as a possible factor related to MS. This theory is referred to as the gut-brain axis thesis. Several studies have shown that MS patients have gut dysbiosis [12]. The gut microbiota is actively involved in the regulation of proinflammatory plasticity and intestinal T lymphocyte (T cell) activity [13]. There is still no specific intestinal microbial strain associated with MS [14], although some authors point out that a reduction in the biodiversity of the intestinal microbiota is observed as a reduction in the number of lactobacilli strains [15].

These imbalances are linked to increased proinflammatory cytokines and general inflammation [16, 17].

Diet has become the most influential factor determining the composition and role of the gut microbiota [18–20]. Restoration of the microbial population in patients with relapsing multiple sclerosis helps to decrease inflammatory events and reactivate the immune system [21].

Modulation of the gut microbiota as therapeutic support in MS is an intervention under study to elucidate whether there are beneficial effects [22]. The more diverse the diet, the more diverse the microbiome and the more adaptable it is to alterations. Unfortunately, dietary diversity has been lost in the last 50 years [23].

Swank and Goodwin pointed out in their research that saturated fatty acids may represent proinflammatory dietary factors with negative effects on MS progression. Their persistent intake leads to dysbiotic gut microbiota [24].

A high intake of saturated fats, sugars, and animal proteins can stimulate the proliferation of specific species of pathogenic bacteria in the intestine. Similarly, it can induce enteric inflammation, damage to the intestinal barrier, and an increase in cross-reactive cells of adaptive immunity. In addition, diet-induced low biodiversity of the gut microbiota is associated with metabolic changes and increased markers of inflammation [25].

Intermittent fasting (IF) seems to confer protection on the central nervous system (CNS) autoimmunity by modifying the gut microbiota. It has a powerful immunomodulatory effect that is partially mediated by the gastrointestinal microbiome [26].

#### Alteration of the intestinal barrier (IB) in MS

In addition to its basic function in the regulation of homeostatic processes, IB contains the immune system of the intestinal mucosa. Gastrointestinal disorders with rupture of the IB show a correlation to CNS demyelination. The intestinal microbiome accessing the circulation may influence the functions of the CNS microglia. Disease-modifying drugs commonly used in MS may modify the intestinal barrier and microbiome [27].

Intestinal dysbiosis as a consequence of Western diets causes intestinal inflammation and a permeable intestinal barrier. The IB must be impermeable mainly to the molecules of the diet that are not fully digested, that is why it exists. Microbial molecules and cells, undigested dietary molecules, and immunocompetent cells can escape from the intestine when the barrier is broken [28].

#### Immune response and oxidative stress, and diet in MS

##### Nutrition as a potential cofactor affecting the inflammatory cascade

Three aspects of MS pathophysiology seem to have the potential to affect disease outcomes. 1) Modify the inflammatory state: Dietary factors that can induce differentiation and proliferation in immune regulatory cells could reduce the formation of new inflammatory lesions using pathways similar to traditional disease-modifying therapies. 2) Protect against neurodegeneration: Dietary factors that dampen CNS inflammation or prevent oxidative stress may prevent chronic demyelination and axonal/neuronal damage. 3) Promote injury repair: Dietary factors may influence axonal remyelination [3].

##### Immune response

Different immune responses, including the adaptive and innate immune systems, are seen in various stages of MS. Immuno-dependent cell death of oligodendrocytes and neurons, and oxidative stress-induced tissue damage, contribute to the pathology of MS [29].

A dynamic balance regulated by the intestinal microbiome, between T helper-17 (Th17) and regulatory T cells (Tregs), is frequently found at intestinal barriers, where they function to protect the host from pathogenic microorganisms and to curb excessive effector T cell responses, respectively. Th17 cells have recently been identified as a unique subset of CD4 + T cells, characterized by the production of IL-17 that promotes tissue inflammation. Treg cells have been identified as specific suppressors of various immune responses and inflammation, and as central guardians of peripheral tolerance. Tregs are generated in both the thymus and the periphery [30].

Experimental MS models have reported that Treg cells have deficits in their function as a result of the aberrant composition of the intestinal microbiota [31, 32]. The increase in the frequency of Th17 cells is correlated with high MS activity [33]. Microbial metabolites are also important, which can cross the blood–brain barrier (BBB) and mediate its effects directly on immune cells within the central nervous system (CNS) or by stimulating pro-inflammatory cytokines, which in turn regulate the autoimmune response in the CNS [34].

Immunologically, MS is correlated with Treg dysfunction, increased Th1 and Th17 responses, increased IL-1, -6, -17, IFN-γ, and TNF-α, and overactivity of autoreactive B cells [16].

Shared functions of vitamin A and vitamin D include enhancement of tight-binding proteins, suppression of IFN-γ and IL-17, and induction of Tregs [35, 36]. Vitamin D deficiency leads to the breakdown of the intestinal barrier, intestinal dysbiosis, and intestinal inflammation [24, 28]. The impact of Vitamin D on MS patients may be mediated by improving the efficiency of the DNA repair system [37].

Fatigue scores were correlated with interleukin (IL)-6 and TNF-α levels [38, 39]. The level of anti-inflammatory cytokines including Interleukin 4 (IL-4) that is produced from Th2 cells decreases in MS [40]. Serum IL-4 levels were higher in RR-MS patients with mild disability compared to those with moderate and severe disability [41]. There are published clinical trials with different kinds of diets that follow the evolution of this indicator in patients with multiple sclerosis [42, 43]. In Mousavi et al., RCT, a Modified anti-inflammatory diet could increase IL-4 levels [42].

##### Oxidative stress

Oxidative stress increases inflammation, causing damage to the myelin sheath and death of neurons in MS patients. The natural evolution of MS is characterized by the secretion of many inflammatory and oxidative stress mediators, including cytokines, such as IL-1β, IL-6, IL-17, TNF-α, and INF-γ. The process of demyelination involves mainly the action of macrophages, B-cells, T cells, and the increased permeability of the BBB [44].

Oxidative stress associated with inflammation and neuronal damage results in the oxidation of cellular components, such as proteins, lipids, and nucleic acid enhancers. This leads to a cycle that can accelerate disease progression [45].

Antioxidants such as melatonin, Vitamin D3, omega-3 polyunsaturated fatty acids (PUFAs), and polyphenol compounds have potential protective effects on MS neurodegeneration [25, 44, 46].

Probiotic intake markedly improved insulin resistance and inflammatory and oxidative stress markers [47].

#### Dysregulated intestinal homeostasis food intolerance and food allergy in MS

The CLIMB study evaluated the association between a self-reported history of allergic conditions with the MS clinic and magnetic resonance imaging activity. The rate of the cumulative number of attacks was 1.48 times higher in patients with MS and food allergies compared to unknown allergies, and the probability of having gadolinium-enhanced lesions on magnetic resonance imaging (MRI) was more than twice [48].

Constant environmental stimulation of the intestine can be both dangerous and beneficial. The balance generated is critical to homeostasis. The gastrointestinal tract contains more lymphocytes than any other tissue compartment, and antigen-presenting cells with specialized functions. The immune system located in the intestine generates a potent T cell-mediated hypo-responsiveness when an antigen contacts initially these cells in the gut, which is called oral tolerance. Food allergy occurs when this system fails [49].

#### Obesity

There is a consensus that obesity is associated with a higher risk of suffering MS in young adulthood, particularly from ages 18–25. Data obtained in Childhood show that eliminating obesity in this population would prevent around 15% of MS cases [50, 51]

Leptin promotes autoreactive T-cell proliferation and proinflammatory cytokine secretion, but inhibits Treg-cell proliferation [52]. Higher levels of leptin and FABP4 and lower adiponectin have been found in pediatric obese MS patients compared to healthy controls. Higher levels of adiponectin were associated with a lower hazard of relapse [53].

Obesity alters the balance between proinflammatory and suppressive T cell responses in adipose tissue, and Tregs lose their phenotypic identity and function [54], resulting in a breakdown of self-tolerance [13, 55]. Improved nutrition education can help people with MS to make healthy dietary changes for weight loss or nutritional purposes [56].

### 2. Evidence from nutrient-focused studies

The potential ways of dietary treatment in pwMS based on the supplementation or administration of nutrients, has been reviewed in multiple articles for years [3, 25, 46].

Food could affect the course of inflammatory diseases. In theory, dietary factors may influence on inflammation, neuroprotection, and repair in MS [3, 46].

We have discussed throughout the background the potential of certain nutrients and supplements in patients with MS, which act on oxidative stress maintaining the homeostasis of the CNS, improving the intestinal microbial balance and regulating the composition of gut microbiota, or in the modulation of the components of inflammatory cascade, and factors with negative effects on the course of MS as pro-inflammatory dietary factors.

Some systematic reviews and meta-analyses that provide evidence about nutrients and supplementation in MS are shown below:

The Cochrane systematic review by Parks et al., 2020, with 30 randomized controlled trials (RCT) and controlled clinical trials (CCT), that analyzes any dietary intervention for MS with the exception of vitamin D, concluded that there was insufficient evidence to determine whether supplementation with antioxidants (beta-carotene, ascorbic acid, alpha-tocopherol, selenium, and polyphenols such as curcumin and quercetin) or other dietary interventions—PUFAs include omega-6 fatty acids and omega-3- fatty acids, biotin in high doses (300 mg per day), cobalamin (vitamin B12)—have some impact on MS-related outcomes in the dysbiotic intestinal microbiota and in low-grade endotoxemia [4].

The findings of the meta-analysis Cochrane by Jagannath et al., 2018 (12 RCTs and quasi‐RCTs. Vitamin. D administered as monotherapy or in combination with calcium/placebo), suggested that vitamin D seemed to have no therapeutic effect on the Expanded Disability Status Scale (EDSS) score, or annualized relapse rates (ARR) in MS patients at the doses used [57].

The systematic review and meta-analysis by Moosazad et al., 2021, using cross sectional and cohort studies, showed that the disability scales decrease with increasing the vitamin D concentration [58].

The systematic review of AlAmmar et al., 2019, analyzed 7 studies -RCT, cohorts and case control studies- and showed that Omega-3 and fish oils supplementations have beneficial effects on reducing the relapsing rate, inflammatory markers, and improving the quality of life for MS patient [59]. On contrast, other systematic review (Sedighiyan et al. 2020) that analyzed 4 RCT, showed that omega-3 supplementation may not have a clinically considerable impact on EDSS or proinflammatory markers [60].

The results of the systematic review of Jiang et al., 2021 -Preclinical trials and meta-analysis of RCT-, showed significant beneficial effects on EDSS scores, and indicated that probiotics may have beneficial effects in the prevention and treatment of MS, with very low certainty of evidence [47].

### 3. Dietary restriction

Chronic or intermittent dietary restriction induces changes in the composition of the intestinal microbiome and metabolites production and their impact on the underlying functional mechanisms [61, 62]. Choi et al., report preliminary data suggesting that a fasting-mimicking diet (FMD) or a chronic ketogenic diet are safe, feasible and potentially effective in the treatment of relapsing remitting multiple sclerosis (RRMS) patients [63].

Large randomized controlled dietary trials find adherence to diet a major hurdle. Patients adapt more easily to a time-restricted diet than to caloric restriction [64].

According to the Thomsen et al., 2018 systematic review, there is not yet strong evidence to say whether gluten plays a role in MS [65]. Passali et al.´s in 2020 reviewed the information available on the feasible involvement of gluten in multiple sclerosis [66].

There is a lack of strong and consistent evidence for dietary interventions in people with MS, demonstrating any effect on key outcomes of MS progression. More robust studies focusing on foods rather than nutrients are needed to strengthen the evidence [67]. At present, it is necessary to provide objective information to the patient, taking into account the growing importance of some interest points in the origin and maintenance of the disease, such as nutrition as a possible cofactor influencing the inflammatory cascade, gastrointestinal symptoms, the state of the microbiota and intestinal barrier, allergies and individual food intolerances or obesity in MS patients.

Nine out of ten pwMS experience fatigue on a regular basis [68]. Fatigue is hard to treat and affects largely PwMS quality of life [69]. The influence of diet on variables such as fatigue and quality of life in multiple sclerosis is of increasing interest [4, 7].

We aim to provide an objective synthesis on the evidence for efficacy and safety of specific diets in MS patients through a rapid review, and meta-analyses of randomized controlled trials (RCTs) examining biomarkers such as Modified Fatigue Impact Scale (MFIS), quality of life (QOL) and Expanded Disability Status Scale (EDSS), and severe adverse events associated with dietary interventions.

## Methods

### Search methods for identification of studies

A rapid systematic search was performed on PubMed and EMBASE up to December 2021 (Additional file 1: Appendix 1. MEDLINE search strategy, Additional file 2: Appendix 2. Embase search strategy). The Cochrane handbook, and the preferred reporting items for systematic reviews and meta-analyses (PRISMA) supported the review [70]. Grey literature, and older articles were excluded. PROSPERO (International prospective register of systematic review. https://www.crd.york.ac.uk /PROSPERO), did not allowed registration of the reviews after data collection (2021).

### Criteria for considering studies for this review

They are shown in Table 1.

**Table 1:** Eligibility criteria used for literature search and screening

*Population*	*PwMS (RR-MS, PP-MS, SP-MS) adult individuals (all types in terms of age, sex, MS duration, disability degree)*
*Intervention*	Diet
*Control*	Placebo/other control interventions
*Outcome*	Disease activity, quality of life, relapse rate, disability, fatigue, adverse events
*Study types*	Randomized controlled trials
*Language*	English

### Outcomes measures

#### Change in Expanded Disability Status Scale (EDSS)

EDSS ranges from 0 (no neurologic abnormality) to 10 (death due to MS). Disability progression is defined as an increase of ≥ 1 point in EDSS if baseline score < 5.5 and of ≥ 0.5 points in EDSS if baseline score ≥ 5.5 [71, 72].

#### Participant reported outcomes

Change in Health‐related quality of life, characterized by the Multiple Sclerosis Quality of Life‐54 (MSQoL‐54) scale [73], and Fatigue, by the Modified Fatigue Impact Scale (MFIS) [74].

#### Safety

Number of severe adverse events associated with dietary interventions within the follow-up period.

### Data extraction

Two researchers independently assessed the articles. In the case of disagreement, a third reviewer was consulted.

### Statistical analyses

Comprehensive meta-analysis (CMA) RStudio 4.1.3.- was used for statistical analyses [75]. We have used the Wan application to estimate the mean and standard deviation from the sample size, median, and/or interquartile range [76].

A random effects model was used to compare diet intervention and control effects, pooling Weighted Mean Difference (WMD) with 95% Confidence Intervals (CI). In several studies, the authors reported the median and interquartile range (IQR), for EDSS, MFIS and QOL comparisons, which we transformed to mean and standard deviation (SD), using the Wan application [76].

### Risk of bias assessment

The score of the studies analyzed on the Jadad scale obtained values of 2–3 out of 5. This is mainly due to lack of blinding of participants and investigators (performance bias) -Oxford quality scoring system (the Jadad scale)- [77]. Two reviewers made this judgment independently.

## Results

**Fig. 1:**
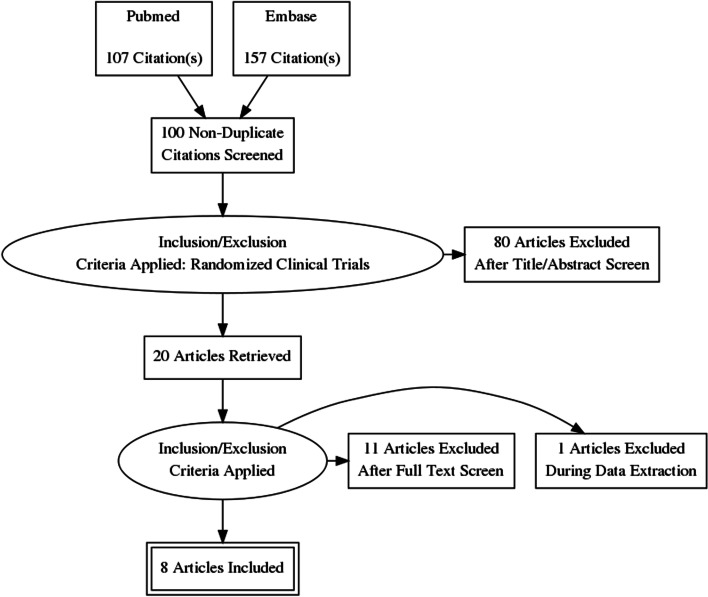
PRISMA Diagram

We have found necessary to perform these meta-analyses investigating the effect of full diets on outcomes related to MS. The method for selecting the studies is shown in Fig. 1. Two reviewers made this judgment independently.

### Limitations

The meta-analyses carried out, evaluated heterogeneous studies with different dietetic interventions. Lack of blinding of participants and investigators (performance bias) was observed in the studies analyzed (the Jadad scale).

Not all variables analyzed were included in all the studies. Adherence and / or body mass index (BMS), as well as basal metabolism, were not controlled in some of them. As we can see in Table 2, the studies have different duration, the longest was the one carried out by Yadav et al. (12 months) [78].

**Table 2:** Studies for Meta-analyses investigating the effect of dietary interventions on MS-related outcomes. 2021

Reference Country	Duration months	Basal characteristics of subjects in control (C) and intervention (I) groups					Harris-Benedict	Intervention/control Restrictions	Energy: protein/ carbohydrate/. fat %
		**Participants total;** **Female %**	**Mean age** **(years) ± SD**	**Mean disease duration (years) ± SD**	**EDSS**	**BMI (Kg/m**^**2**^**)**			
Mousavi-Shirazi-Fard 2020 [42]. Iran	3	100 RRMS F (87%) I:50, C:50	I:35,2 ± 6,61 C:36,26 ± 7,23	I:6,61 ± 2,88 C:5,74 ± 2,7		I:24,40 ± 2,96 C:24,10 ± 3,11	Yes. Activity factor: 1,2–1,3	Modified anti-inflammatory diet / control: (WHO) healthy diet Restrictions.: sucrose, processed food, fast food, fried food, and animal fat. Substitutions: white rice /brown rice, white bread/whole wheat bread, high fat dairy/probiotics low fat products	15% / 55% / 30%
Katz Sand 2019 [79] Iowa USA	6	36 MS (28RRMS, 3SPMS,1PPMS) F (100%). I:18, C:18	I:44(37–51) C:41(30–49)	I:5,4(2–10,7) C:4,8 (2–11,2)	I:2(0–3) C:2(0–5)	I:26(25–37) C:25(23–30)		Modified Mediterranean dietary program (KS-mMdiet) / control: Western style diet that include at least meat or dairy Restrictions: meat, dairy, white grains and processed food, salt intake to 2 g/day, and not eating for at least 12 h per night	-
Choi 2016 [63]. Germany	6	48 RRMS F (79%) FMD:18, KD: 18, C:12	FMD:44,4 ± 11,1 KD:41,3 ± 8,2 C:50,5 ± 10,4	FMD:11 ± 7,7 KD:6,3 ± 4,3 C:9,9 ± 9,2	FMD: 4 (2,4–4) KD: 3(2,4–3,5) C: 2,5 (1,5–4)	FMD:26 ± 4,8 KD:26,9 ± 5,3 C: 27,3 ± 6,9		Fasting-Mimicking-diet for 7 days, followed by a Mediterranean diet for 6 months (FMD)/ Low Glycemic Load Ketogenic Diet (KD) / control: Criteria of a regular diet in German population	KD < 50 g carbohydrates, > 160 g fat and < 100 g protein intake daily
Irish 2017 [80]. USA	3	17 RRMS F (88%) I:8, C:9	I:35.4 ± 5.7 C:37.1 ± 3.7	*P* = 0,26				Modified Paleolithic diet. Gluten Free / control: typical physician recommendation for MS Restrictions: complete abstinence from products containing gluten, dairy, potatoes, and legumes	
Rezapour‐Firouzi 2013 [43] Iran	6	65 RRMS F (65%) A:23, B:22, C:20	A:34,2 ± 7,5 B:35,9 ± 7,8 C:33,7 ± 7,8	A:6,26 ± 3,9 B: 7,55 ± 5,08 C:6,6 ± 4,0	A:2,76 ± 1,39 B:3,45 ± 1,41 C:3,25 ± 1,9	**-**		A: Co-supplemented hemp seed and evening primrose oils and advised hot nature diet. B: Supplement olive oil C: Co-supplemented hemp seed and evening primrose oils Restrictions: low intake of cholesterol, hydrogenated or trans fatty acids and saturated fats reducing sugar and refined starch, dairy products with honey or date and eliminating foods with Cold nature	
Yadav 2016 [78]. USA	12	61 RRMS F: (93,3%) I:32, C:29	C:40.9 ± 8.48 I:40.8 ± 8.86	C:5.3 ± 3.86 I:5.33 ± 3.63	I:2.72 ± 1.05 C:2.22 ± 0.90	I:28,4 ± 6,76 C:29,3 ± 7,42		Very low fat plant based Diet / Control group: typical physician recommendation for MS Restrictions: Meat, fish, eggs, dairy products, and vegetable oils such as corn and olive oil were prohibited	14% / 76% / 10%
Bohlouli 2021 [81]. Iran	6	147 RRMS F: (83%) I:68, C:79	I:38,6 ± 8,4 C:40,0 ± 9,6	I:8,1 ± 5,7 C:9,3 ± 6,9	I:1,7 ± 0,7 C:2,0 ± 0,9	I:26,1 ± 4,1 C:25,9 ± 4,5	Yes. To calculate the BEE	Modified Mediterranean diet:/ Traditional Iranian diet Restrictions: low to moderate consumption of dairy products, meat and poultry. It is recommended whole grains and monounsaturated fatty acids	17% / 51% / 32%
Platero 2020 [82] Spain	4	51 MS I:27, C:24	(median) I:45 C:50	(median) I:9 C:13,5	I:3,37 ± 2,03 C:3,8 ± 2	I:25,92 ± 5,29 C:25,87 ± 6,10		Mediterranean-type food pattern*, with extra virgin coconut oil and supplemented with Epigallocatechin gallate (EGCG) / Control group: the same isocaloric diet and placebo Restrictions: detriment of meat and meat products. It is recommended whole grain bread	20%/40%/40%

*I* Intervention group, *C* Control group, *F* Females, *SD* Standard deviation, *QoL* Quality of life, *RRMS* Relapsing remitting multiple sclerosis, *SPMS* Secondary progressive multiple sclerosis, *PPMS* Primary progressive multiple sclerosis, *BEE* Basal energy expenditure

#### Recording variables

Only Bohlouli [81], Mousavi [42] and Yadav [78] studies showed MFIS data (Table 4). Many studies showed improvements in fatigue with different variables: fatigue severity scale-FSS- (Irish [80], Yadav [78]); or Neurological fatigue index NFI- (Katz Sand) [79].

In Yadav study [78], we used MFIS 25 and MFIS 5 as equivalent variables (Crombach´s alpha coefficient for both questionnaires was 0,81 and 0,80, respectively, suggesting internal consistency), it was necessary to adapt the scales, MFIS 21 (score range 0–84) and MFIS 5 (score range 0–20).

We have used the Wan application to calculate the mean and standard deviation from the sample size, median, and/or interquartile range [76].

## Results

Initially, nine studies met inclusion criteria. We excluded Lee study, with 14 MS patients (Modified Paleolithic diet-5 / MCT Based Ketogenic Diet-4 / control: usual diet-4), due to difficulty in obtaining values of the analyzed variables, in this publication [83]. Selected studies are shown in Table 2.

Results from four meta-analyses (EDSS, MFIS, MQoL, PhQoL) are shown in Tables 3, 4, 5 and 6. Not all the studies included all the variables contemplated in the meta-analysis. Two reviewers made this judgment independently.

**Table 3:**
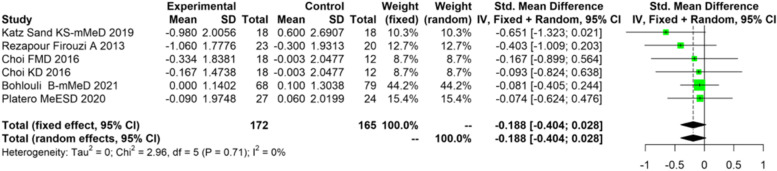
Meta-analysis of studies on diet in multiple sclerosis containing information on EDSS

**Table 4:**
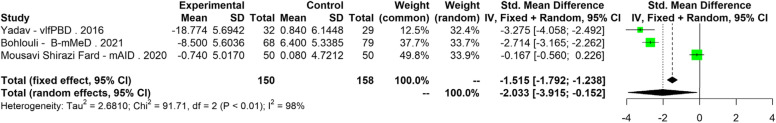
Meta-analysis of studies on diet in multiple sclerosis containing information on MFIS

**Table 5:**
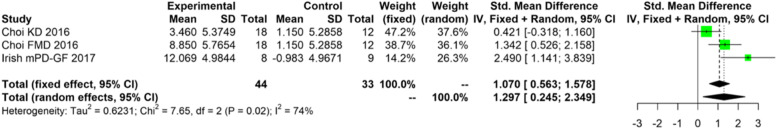
Meta-analysis of studies on diet in multiple sclerosis containing information on Physical MSQOL

**Table 6:**
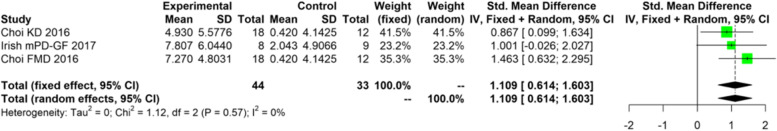
Meta-analysis of studies on diet in multiple sclerosis containing information on Mental MSQOL

### EDSS

Table 3 six different studies were analyzed (337 patients studied). The test of heterogeneity shows us the values distributed homogeneously, therefore we use the model of fixed values. The difference between means (SMD) of the control group and the intervention group was -0.1879, 95%-IC (-0.436,0.0279), *p*-value of 0.0878.

### MFIS

Table 4 three different studies were analyzed (308 patients studied), (Bohlouli [81], Yadav [78] and Mousavi [42] studies). The test of heterogeneity shows us the values distributed heterogeneously. SMD of the control group and intervention group was -2,033, 95%-IC (-3,195, -0,152), *p*-value of 0.0341.

### MSQOL-physical (MSQOL-P)

Table 5 two different studies were analyzed (77 patients studied), (Choi [63], and Irish [80] studies) The test of heterogeneity shows us the values distributed heterogeneously. SMD 1.297, (0.2454,2.3485). *p*-value of 0,01.

### MSQOL-mental (MSQOL-M)

Table 6 three different interventions were analyzed (77 patients studied), (Choi [63], and Irish [80] studies). The test of heterogeneity shows us the values distributed homogeneously. We obtain SMD 1.1086, 95%-CI (0.6143,1.6029). *p* < 0,0001.

### Adverse events

The studies (eight different studies were analyzed) found no severe diet-related adverse events (diarrhea, abdomen pain, constipation, appetite changes).

### Adherence

Different methods were used to monitor adherence, all based on self-reported mean adherence: Yadav [78], used the percentage of saturated fat; Mousavi [42] and Bohlouli [81], the dietary inflammatory index (DII) score; and Irish [80], gluten and dairy consumption.

Bohlouli traditional Iranian diet (TID) plan was adjusted for energy intake to avoid unexpected body weight changes. Bohlouli showed relationship between the DII and MFIS. DII predicted MFIS in the modified Mediterranean diet (mMeD) group [81].

The Yadav [78] and Mousavi [42] studies looked at BMI scores. Mousavi using modified anti-inflammatory diet, found no change in BMI scores between the control and intervention groups over the study period. However, Yadav, using a very low-fat vegetable diet, found a significant relationship between dietary intervention, weight loss, and MFIS (weight loss accounting for 42.5% of the total effect of diet on MFIS).

Among the various diets investigated, the modified Mediterranean diet is easier to be maintained (Mousavi diet adherence: 96,15% [42], Katz Sand: 90,3% [79]), compared to restrictive diets (Irish: 50% [80], Bohlouli: 75,5% [81]).

## Discussion

We have considered necessary these Meta-analyses investigating the effect of whole diets on MS-related outcomes, using RCTs, because there are few ongoing RCTs in MS with specific diets versus control, only the study “Low Fat Diet for Fatigue in MS” (Yadav. Clinicaltrials.gob) was found. Furthermore, in the scientific reviews up to 2019, it is emphasized the insufficient evidence to recommend the use of a specific diet for people with MS, because most of the human trials have been small, without a control group, and not blinded, which limits their generalizability. Many have also been short-lived, which could limit the ability to find clinically significant changes [4, 84]. There is very little mention of PP-MS, where nutritional intervention is particularly important.

In the last few years, publications support the importance of some interest points in the origin and maintenance of the disease, such as nutrition, having a role in influencing the inflammatory cascade, gastrointestinal symptoms, the state of the microbiota and intestinal barrier, allergies, and individual food intolerances.

It is difficult to reach level of evidence 1 +  + in dietary studies, in order to make recommendations following SIGN (Scottish Intercollegiate Guidelines Network) criteria [85]. This is mainly due to lack of blinding of participants and investigators (performance bias), lack of dietary adherence in the studies, baseline patient characteristics differing between studies, not inclusion of basal metabolism to calculate dietary needs in some of the studies analyzed, disregard of patient feedback and the wide range of dietary interventions. Moreover, the studies analyzed in these meta-analyses have been done in different countries (Iran, USA, Germany …), with different nutrition profiles (different microbiota). Also, the body mass index of the patients varies in the different studies.

On the other hand, the potential of dietary interventions is considerable, and presumably the benefit / risk ratio is skewed in their favor. Benefits should always be weighed against the risks, burden and costs associated. The unique situation of patients also should be considered, including their goals and preferences [86]. In the studies analyzed in these meta-analyses, no adverse effects related to diet have been reported.

The accumulated evidence from these meta-analyses is not sufficient to support that disease-associated disability, as measured by EDSS, is modified by dietary changes. However, different variables associated with quality of life and fatigue did show improvement after dietary intervention in people with MS.

The reason for dietary intervention is its effect on the control of the inflammation process and oxidative stress. [28]. The studies showing the best results (Yadav [78], Bohlouli [81], Katz Sand [79], and Rezapour [43]), have in common the dietary exclusion or low consumption of saturated fats, white flour, dairy and sugar; these studies also propose an exclusion or reduction in meat consumption and the intake of sufficient fruits, vegetables and fish. Likewise, a 12-h fasting at night brings good results [79].

Katz Sand [79], and Yadav [78] diets advise against the intake of meat. Rezapour [43] and Bohlouli [81], limit meat (low intake of cholesterol, hydrogenated or trans fatty acids and saturated fats). The Bohlouli [81], and Mousavi [42] studies have also in common the analysis pre-post intervention of DII score.

A causal relationship between diet and fatigue was predicted by Yadav, taking into account preliminary analyzes, with changes in BMI, total serum cholesterol and insulin levels. [78]. Yadav had the better MFIS results among the meta-analysis studies. The longer duration of the study (12 months) and the weight loss achieved could have an additional effect in this sense compared to the studies by Bohlouli [81] -6 months-, and Mousavi [42] -3 months-. In the latter two studies, no weight loss was observed. Furthermore, in the Bohlouli study, the diets of control and intervention group were adjusted for energy intake, to avoid unexpected body weight changes [81].

In the studies by Bohlouli [81] and Mousavi [42], the MFIS in the intervention group decreased significantly while the DII score decreased, clearly showing the potential of anti-inflammatory diets.

Fatigue scores were correlated with proinflammatory cytokines such as IL-6, and with TNF-α levels [38]. Only Mousavi [42] and Rezapour [43] studies found increased interleukin 4 (anti-inflammatory cytokine) in the intervention group. Future evidence will be provided through the measurement of different clinical variables such as inflammatory biomarkers, including IL-17, IL-4, and highly sensitive C-reactive protein [42]. CHI3L1 (chitinase-3-like protein), GFAP (glial fibrillary acidic protein), and NFL (neurofilament light chain) [87], among others.

### Gluten-free diets

There are already clinical trials, such as the one by Whals (the Waves randomized parallel-arm clinical trial) [88], with the modified paleolithic diet, that show that eliminating gluten, casein, and lecithin from the diet, benefits RRMC patients with fatigue, MFIS improves (-9,87 + -1,93 *p* < 0,0001y QoL) and confirms the results of the studies of Irish et al. [80], and Lee [83], RCTs that also eliminate gluten from the diet in MS. Among the exclusion criteria of the Wahls´s study [88] was of patients with celiac disease. Lee [83], and Irish [80] studies excluded patients with gluten-free diets. These 2 studies do not specifically select non-celiac patients. Among other characteristics, the three diets have in common the exclusion of gluten, milk and the control of sugar intake. Wahls recommends using the modified Paleolithic diet for fatigued patients with RRMS. The Waves clinical trial, get improvements in EDSS and MFIS [88].

Swank and Wahls diets contain a lower proportion of some nutrients compared to those needed in the usual diet, especially magnesium supplements and vitamins A, C, D and E. Deficiency of these micronutrients should be controlled [89].

Usually, the MS patient does not receive specific instructions on diet or supplements [90]. However, there is a growing interest on the part of patients in eating habits, as well as a large amount of information on dietary interventions on the Internet. The scientific evidence supporting this information is scant. Researchers and care professionals should be up to date on popular MS diet strategies [7], and their potential, given the lack of high-grade evidence of these interventions. It is important to update the importance of diet in MS by the professionals involved. Patients prefer information on diet provided by neurologists [6]. Nutritional counseling about the diet for small groups within several sessions is also advisable to improve knowledge of patients and resolve doubts [79].

Regarding different baseline characteristics of patients, there are no validated tests that identify food intolerance. Likewise, there is a lack of training for clinicians on the influence of microbiota in autoimmune diseases. The regulation of the gut microbiota is important in MS patients [22]. Avoiding obesity should be considered a priority objective in MS, and dietary interventions through multidisciplinary teams should be recommended [56]. Diet cannot be generalized in pwMS, but it is essential to control the diet of pwMS, and adapted it to their caloric needs, microbiota and intolerances.

New systematic reviews and clinical trials (randomized parallel-arm clinical trial) are underway that will help in the selection of appropriate diets.

## Conclusions

It is difficult to reach a sufficient level of evidence in dietary studies, to make recommendations. We should consider a new practice when evaluating the degree of recommendation for decision-making about diet in MS mainly due to the peculiarities of the dietary interventions.

These meta-analyses cumulative evidence support the association of dietary interventions with a tendency to reduce fatigue and an increase in QoL among MS patients included in these studies and encourages us to know the diet of pwMS and to take action to improve it.

Considering the potential of dietary interventions and the suitable benefit/risk ratio, neurologists must be conscious of the great importance of making interventions on diet in MS, keeping in mind the expected adherence of the patient, and the need for multidisciplinary work to empower the patient and achieve results.

There are dietary interventions with some evidence of benefit for patients with MS, which could be chosen based on adherence, patient preferences and individual outcomes. Based on the available literature, it seems appropriate to adapt the diet to caloric needs by controlling weight, monitoring the microbiota, assessing the need for probiotics, vitamin D and omega-3 fatty acids, and detecting food intolerances.

Unfortunately, there are still no precise concrete recommendations on a specific dietary plan diet for MS patients. In this sense, large prospective clinical trials are needed. New systematic reviews and randomized parallel-arm clinical trials are underway that will help in the selection of appropriate diets.

## Supplementary Information

**Additional file 1: Appendix 1.** MEDLINE search strategy. Meta-analysis/Randomized controlled trial/Systematic review.

**Additional file 2: Appendix 2.** EMBASE search strategy.

## Data Availability

All data generated or analyzed during this study are included in this published article.

## Abbreviations

ARR: Annualized relapse rates
BBB: Blood brain barrier
B-mMeD: Bohlouli modified mediterranean diet
BMS: Body mass index
CI: Confidence Interval
CMA: Comprehensive meta-analysis
CNS: Central nervous system
CSF: Cerebrospinal fluid
DII: Dietary inflammatory index
DNA: Deoxyribonucleic acid
EBNA: Epstein-Barr Nuclear Antigen
EBV: Epstein-Barr virus
EDSS: Expanded disability status scale
FMD: Fasting-mimicking diet
GI: Gastrointestinal
IB: Intestinal barrier
IBD: Inflammatory bowel disease
IF: Intermittent fasting
IFN: Interferon
IL: Interleukin
KD: Ketogenic diet
KS-mMdiet: Katz-Sand modified Mediterranean dietary program
LPS: Lipopolysaccharides
M cells: Microfold cells
mAId: Modified anti-inflammatory diet
meESD: Mediterranean extra virgin coconut oil diet
MFIS: Modified Fatigue Impact Scale
MRI: Magnetic resonance imaging
MS: Multiple sclerosis
MSQOL: Multiple Sclerosis Quality of Life
MSQOL-M: MSQOL-mental
MSQOL-P: MSQOL-physical
PP-MS: Primary progressive multiple sclerosis
PUFAs: Polyunsaturated fatty acids
pwMS: People with multiple sclerosis
RCT: Randomized controlled trial
RR-MS: Relapsing–remitting multiple sclerosis
SMD: Difference between means
SP-MS: Secondary-progressive multiple sclerosis
T-cell: Lymphocytes T
Th17 cells: Lymphocytes T helper-17 cells
TID: Traditional Iranian diet
vlfPBD: Very low-fat plant-based Diet
WMD: Weighted Mean Difference
