# Supplementary material for: Efficacy of diet on fatigue, quality of life and disability status in multiple sclerosis patients: rapid review and meta-analysis of randomized controlled trials
Source: BMC Neurol. 2022 Oct 20;22:388. doi: 10.1186/s12883-022-02913-w (PMC9583472; doi:10.1186/s12883-022-02913-w)
Supplement: Supplementary file 1 — Additional file 1: Appendix 1. MEDLINE search strategy. Meta-analysis/Randomized controlled trial/Systematic review. [file 12883_2022_2913_MOESM1_ESM.docx]

| Appendix 1. MEDLINE search strategy. Meta-analysis/Randomized controlled trial/Systematic review |  |  |
| --- | --- | --- |
| MEDLINE search strategy | **Period** | **Number of studies** |
| ((multiple sclerosis) AND (diet)) AND (edss) | 1999-2022 (08/01) | 13 |
| ((multiple sclerosis) AND (diet)) AND (mfis) | 2016-2022 (08/01) | 2 |
| ((multiple sclerosis) AND (diet)) AND (quality of life) | 2005-2022 (08/01) | 9 |
| ((multiple sclerosis) AND (diet)) AND (msfc) |  | 0 |
| ((multiple sclerosis) AND (diet)) AND (fss) |  | 5 |
| ((multiple sclerosis) AND (diet)) AND (nri) |  | 0 |
| ((multiple sclerosis) AND (diet)) AND (relapse) |  | 33 |
|  |  |  |
| ((relapsing remitting multiple sclerosis) AND (diet)) AND (EDSS) | 2013-2022 (08/01) | 7 |
| ((relapsing remitting multiple sclerosis) AND (diet)) AND (mfis) | 2016-2022 (08/01) | 1 |
| ((relapsing remitting multiple sclerosis) AND (diet)) AND (quality of life) | 2005-2022 (08/01) | 4 |
| ((relapsing remitting multiple sclerosis) AND (diet)) AND (msfc) |  | 0 |
| ((relapsing remitting multiple sclerosis) AND (diet)) AND (fss) |  | 1 |
| ((relapsing remitting multiple sclerosis) AND (diet)) AND (nri) |  | 0 |
| ((relapsing remitting multiple sclerosis) AND (diet)) AND (relapse) |  | 23 |
|  |  |  |
| ((primary progressive Multiple sclerosis) AND (diet)) AND (edss) |  | 1 |
| ((primary progressive Multiple sclerosis) AND (diet)) AND (mfis) |  | 0 |
| ((primary progressive Multiple sclerosis) AND (diet)) AND (quality of life) | 2012-2022 (08/01) | 3 |
| ((primary progressive Multiple sclerosis) AND (diet)) AND (msfc) |  | 0 |
| ((primary progressive Multiple sclerosis) AND (diet)) AND (fss) |  | 1 |
| ((primary progressive Multiple sclerosis) AND (diet)) AND (nri) |  | 0 |
| ((primary progressive Multiple sclerosis) AND (diet)) AND (relapse) |  | 4 |
|  |  | 107 |
