# Supplementary material for: Efficacy of diet on fatigue, quality of life and disability status in multiple sclerosis patients: rapid review and meta-analysis of randomized controlled trials
Source: BMC Neurol. 2022 Oct 20;22:388. doi: 10.1186/s12883-022-02913-w (PMC9583472; doi:10.1186/s12883-022-02913-w)
Supplement: Supplementary file 2 — Additional file 2: Appendix 2. EMBASE search strategy. [file 12883_2022_2913_MOESM2_ESM.docx]

| \| **Appendix 2. EMBASE search strategy** \| \| --- \| |  |
| --- | --- | --- |
| EMBASE search strategy | **Number of studies** |
| multiple AND sclerosis AND diet AND edss AND ([cochrane review]/lim OR [systematic review]/lim OR [meta analysis]/lim OR [controlled clinical trial]/lim OR [randomized controlled trial]/lim) AND [1966-2022]/py | 27 |
| multiple sclerosis' AND diet AND mfis AND ([cochrane review]/lim OR [systematic review]/lim OR [meta analysis]/lim OR [controlled clinical trial]/lim OR [randomized controlled trial]/lim) AND [1966-2022]/py | 6 |
| multiple sclerosis' AND diet AND quality AND of AND life AND ([cochrane review]/lim OR [systematic review]/lim OR [meta analysis]/lim OR [controlled clinical trial]/lim OR [randomized controlled trial]/lim) AND [1966-2022]/py | 40 |
| multiple sclerosis' AND diet AND fss AND ([cochrane review]/lim OR [systematic review]/lim OR [meta analysis]/lim OR [controlled clinical trial]/lim OR [randomized controlled trial]/lim) AND [1966-2022]/py | 6 |
| multiple sclerosis' AND diet AND nri AND ([cochrane review]/lim OR [systematic review]/lim OR [meta analysis]/lim OR [controlled clinical trial]/lim OR [randomized controlled trial]/lim) AND [1966-2022]/py | 0 |
| multiple sclerosis' AND diet AND relapse AND ([cochrane review]/lim OR [systematic review]/lim OR [meta analysis]/lim OR [controlled clinical trial]/lim OR [randomized controlled trial]/lim) AND [1966-2022]/py | 22 |
| multiple sclerosis' AND diet AND msfc AND ([cochrane review]/lim OR [systematic review]/lim OR [meta analysis]/lim OR [controlled clinical trial]/lim OR [randomized controlled trial]/lim) AND [1966-2022]/py | 4 |
| relapsing AND remitting AND multiple AND sclerosis AND diet AND edss AND ([cochrane review]/lim OR [systematic review]/lim OR [meta analysis]/lim OR [controlled clinical trial]/lim OR [randomized controlled trial]/lim) AND [1967-2022]/py | 10 |
| relapsing AND remitting AND multiple AND sclerosis AND diet AND mfis AND ([cochrane review]/lim OR [systematic review]/lim OR [meta analysis]/lim OR [controlled clinical trial]/lim OR [randomized controlled trial]/lim) AND [1967-2022]/py | 5 |
| relapsing AND remitting AND multiple AND sclerosis AND diet AND quality AND of AND life AND ([cochrane review]/lim OR [systematic review]/lim OR [meta analysis]/lim OR [controlled clinical trial]/lim OR [randomized controlled trial]/lim) AND [1967-2022]/py | 15 |
| relapsing remitting multiple sclerosis' AND diet AND fss AND ([cochrane review]/lim OR [systematic review]/lim OR [meta analysis]/lim OR [controlled clinical trial]/lim OR [randomized controlled trial]/lim) AND [1966-2022]/py | 1 |
| relapsing remitting multiple sclerosis' AND diet AND nri AND ([cochrane review]/lim OR [systematic review]/lim OR [meta analysis]/lim OR [controlled clinical trial]/lim OR [randomized controlled trial]/lim) AND [1966-2022]/py | 0 |
| relapsing AND remitting AND multiple AND sclerosis AND diet AND relapse AND ([cochrane review]/lim OR [systematic review]/lim OR [meta analysis]/lim OR [controlled clinical trial]/lim OR [randomized controlled trial]/lim) AND [1967-2022]/py | 12 |
| relapsing AND remitting AND multiple AND sclerosis AND diet AND msfc AND ([cochrane review]/lim OR [systematic review]/lim OR [meta analysis]/lim OR [controlled clinical trial]/lim OR [randomized controlled trial]/lim) AND [1967-2022]/py | 2 |
| primary and progressive and multiple and sclerosis and diet and AND edss AND ([cochrane review]/lim OR [systematic review]/lim OR [meta analysis]/lim OR [controlled clinical trial]/lim OR [randomized controlled trial]/lim) AND [1967-2022]/py | 1 |
| primary and progressive and multiple and sclerosis and diet and AND mfis AND ([cochrane review]/lim OR [systematic review]/lim OR [meta analysis]/lim OR [controlled clinical trial]/lim OR [randomized controlled trial]/lim) AND [1967-2022]/py | 0 |
| primary AND progressive AND multiple AND sclerosis and diet and quality and of and life and AND ([cochrane review]/lim OR [systematic review]/lim OR [meta analysis]/lim OR [controlled clinical trial]/lim OR [randomized controlled trial]/lim) AND [1967-2022]/py | 5 |
| primary and progressive and multiple and sclerosis and diet AND fss AND ([cochrane review]/lim OR [systematic review]/lim OR [meta analysis]/lim OR [controlled clinical trial]/lim OR [randomized controlled trial]/lim) AND [1967-2022]/py | 0 |
| primary and progressive and multiple and sclerosis and diet AND nri AND ([cochrane review]/lim OR [systematic review]/lim OR [meta analysis]/lim OR [controlled clinical trial]/lim OR [randomized controlled trial]/lim) AND [1967-2022]/py | 0 |
| primary and progressive and multiple and sclerosis and diet AND relapse AND and and AND ([cochrane review]/lim OR [systematic review]/lim OR [meta analysis]/lim OR [controlled clinical trial]/lim OR [randomized controlled trial]/lim) AND [1967-2022]/py | 1 |
| primary and progressive and multiple and sclerosis and diet and AND msfc AND ([cochrane review]/lim OR [systematic review]/lim OR [meta analysis]/lim OR [controlled clinical trial]/lim OR [randomized controlled trial]/lim) AND [1967-2022]/py | 0 |
|  | 157 |
